# Frequency domain shows: Fall-related concerns and sensorimotor decline explain inability to adjust postural control strategy in older adults

**DOI:** 10.1371/journal.pone.0242608

**Published:** 2020-11-20

**Authors:** Mascha Pauelsen, Hedyeh Jafari, Viktor Strandkvist, Lars Nyberg, Thomas Gustafsson, Irene Vikman, Ulrik Röijezon

**Affiliations:** 1 Department of Health Sciences, Luleå University of Technology, Luleå, Sweden; 2 Department of Computer Science, Electrical and Space Engineering Luleå University of Technology, Luleå, Sweden; University of Minnesota, UNITED STATES

## Abstract

Human postural control is a complex system and changes as we age. Frequency based analyses have been argued to be useful to identify altered postural control strategies in balance tasks. The aim of this study was to explore the frequency domain of the quiet stance centre of pressure of older adults with various degrees of fall-related concerns and sensorimotor functioning. We included 45 community dwelling older adults and used a force plate to register 30 seconds of quiet stance with eyes open and closed respectively. We also measured sensory and motor functions, as well as fall-related concerns and morale. We analysed the centre of pressure power spectrum density and extracted the frequency of 4 of its features for each participant. Orthogonal projection of latent structures–discriminant analysis revealed two groups for each quiet stance trial. Group 1 of each trial showed less sensory and motor decline, low/no fall-related concerns and higher frequencies. Group 2 showed more decline, higher fall-related concerns and lower frequencies. During the closed eyes trial, group 1 and group 2 shifted their features to higher frequencies, but only group 1 did so in any significant way. Higher fall-related concerns, sensory and motor decline, and explorative balancing strategies are highly correlated. The control system of individuals experiencing this seems to be highly dependent on vision. Higher fall-related concerns, and sensory and motor decline are also correlated with the inability to adjust to faster, more reactive balancing strategies, when vision is not available.

## Introduction

Human postural control systems act to maintain balance and body orientation. The central nervous system integrates different modalities of sensory information—from visual, auditory, vestibular, and somatosensory receptors—and creates coordinated motor actions and reactions [1,2]. As people age, their sensorimotor systems deteriorate [3] and their movement ability declines [2,4]. The rate of decline can vary greatly depending on lifestyle, social and genetic factors [5,6]. When muscle strength and somatosensory input decrease and reaction times increase, the risks of falls and developing fall-related concerns (FrC) increase [1,7,8]. It is clear that falls as well as FrC have a great impact on activity and participation levels and the general well-being of older adults [9]. FrC have been described under various names, each describing different concepts within FrC: fear of falling, falls self-efficacy or lack of balance confidence, and consequence concerns. This, and the fact that high FrC is prevalent both among older people with and without fall experiences [10–14] motivates its reconceptualization as a multifactorial and multidirectional phenomenon [15]. This reconceptualisation includes factors such as balance performance, beliefs, activity avoidance and somatosensory decline, which is in line with our recent findings that FrC variance–as measured by the FES-I–is best explained by physical performance, morale and fear rather than fall history [16].

Knowing that a decline in sensorimotor aptness correlates to an increase in FrC, and thus a decrease in physical activity levels, it is important to explore ways to easily identify specific features in human postural control, which correlate to FrC. Clinically, this would lead to a deeper understanding of the underlying sensorimotor systems by means of a quick and feasible postural sway test. This is important for tailored treatment programs that would not only improve balance but possibly also decrease FrC and reduce falls. Successful treatment might lead to higher activity levels and a higher quality of life through active ageing [17].

Posturography has long been, and still is, widely used to describe and analyse human postural control. It comprises of measuring the trajectory of the participant’s centre of pressure (CoP) by use of a force plate. In quiet stance, the CoP trajectories represent the postural sway during the task [18]. Traditional features include trajectory distance, velocity, amplitude, and area of the two dimensional–or spatial–signal in the form of a time series. These traditional spatial features of the time domain of the CoP signals have been used to show the association between declined sensorimotor systems and the variation in FrC, but seem to be too crude to identify specific postural control strategies linked to FrC [19].

The frequency domain of the CoP signals might be a better place to look in an effort to identify the individual sensory and motor systems and control strategies, which together make up human balance [20]. Several methods for decomposing the CoP signal into its various components have been presented in the literature, including fractional Brownian-motion analysis [21] and the slow (rambling) and fast (trembling) components [22] where the slow component is argued to represent sensory input and processing, while the fast component is argued to represent mechanical stiffness, motor commands and possibly feedback based reflexes. Others have concluded that the slow component represents an exploratory sway strategy while the faster component represents reactive postural strategies [23,24]. Multifractal detrended fluctuation analysis can reveal the width of the multifractal spectrum of CoP [25]. It is a method that can differentiate between those with a wider and those with a narrower multifractal spectrum width and suggests that a wider spectrum indicates a less stable postural control [26], but the interpretation of spectrum width values remains uncertain [27]. Through wavelet analysis, different timescales and frequency bands of the signal can be discovered [28]. These different bands are thought to each correspond with different sensory and motor systems. There are, however, some variations in the literature regarding the cut-off frequency values and the exact mechanisms underlying the various frequencies [29–37]. The wavelet transform is a (time) localised method to transform a signal from its time domain to a time-frequency domain [38]. A signal transformation that allows to look at frequencies regardless of where in the signal they appear is the Fourier transform [39]. This method will then allow for an analysis of the amount of power in pre-determined frequency bands or in all separate frequencies (power spectrum density, or psd).

With the uncertainty of how to interpret spectral width or at which frequencies to create bands, we identified the psd method to be the most suitable to explore the relationship between FrC and postural control. To our knowledge, FrC has not been researched in relation to changes in the frequency domain of CoP signals.

The aim of this study was to explore the frequency domain of the quiet stance CoP signals of older adults with various degrees of FrC and sensorimotor functioning.

## Materials and methods

Written informed consent was obtained from all individual participants included in the study. This study design was approved by the Regional Ethical Review Board in Umeå, Sweden (ref no. 2015-182-31) and was conducted according to the principles expressed in the 1964 Helsinki declaration.

### Sample

This cross-sectional study is part of the BAHRT project (Balancing Human and Robot) for which a sample of 153 randomly selected older adults were recruited (70+ years old, community dwelling in the municipality of Luleå, Northern Sweden). The exclusion criteria for the current study were: an MMSE score of 23 or lower (indicating cognitive decline of a level which makes it hard to follow instructions) [40], not being able to perform the walking task in the Short Physical Performance Battery [41] and not being able to read the large print in the MMSE (80pts block letters). After screening the original sample for the exclusion criteria, 126 persons were invited for testing in the movement science laboratory. Of those, 45 accepted to participate. The basic characteristics of the participants are presented in Table 1.

**Table 1:** Characteristics of the participants.

Characteristic (mean ± SD)	All (n = 45)	Women (n = 27)	Men (n = 18)
Age	75.2 ± 4.5	76.0 ± 5.0	73.9 ± 3.3
FES-I	21 ± 4.5	22 ± 4.4	20 ± 4.7
Short Physical Performance Battery	11 ± 2.2	10 ± 2.4	11 ± 1.7
Philadelphia Geriatric Center Morale Scale	14 ± 2.2	13 ± 2.2	14 ± 2.0

### Data

Participants were questioned about FrC using the falls efficacy scale–international (FES-I) instrument. The FES-I measures how concerned one is about falling while performing a range of tasks and is a valid and reliable instrument for this purpose. The scores range from 16 to 64 where a higher score indicates the person is more concerned [42]. As morale had shown to be an important factor in FrC, the Philadelphia Geriatric Center Morale Scale (PGCM) was included in our measurements and statistical modelling. It consists of 17 “yes” or “no” questions about interpersonal and intrapersonal aspects of ageing, surrounding three main factors: agitation, attitude toward own aging, and lonely dissatisfaction [43]. As an indication of the sample’s functional level, their scores on the Short Physical Performance Battery were included in Table 1.

Visual acuity was screened with an NFD vision chart. This chart is used at a distance of 5 m and scored from 0,1 (worse) to 1,0 (normal). Participants were screened for positive vestibular nystagmus with the help of Frenzel goggles. Neck proprioception (joint position sense–JPS) was measured with an 8-camera motion capture system (Qualisys, Sweden) during an active-active cervical rotation repositioning task to neutral head position. Six trials were repeated in left and right rotation, respectively. Absolute error (AE) was calculated as outcome measure. Knee and foot JPS were assessed with the use of a Biodex System 3 machine. The knee repositioning was done at 30° flexion from 90° and the ankle was repositioned to 5° dorsal flexion from 20° plantar flexion, repeated three times. The tests were performed as active-active movement, and AE was calculated as outcome measure. The same Biodex system was used for isometric strength testing of the larger muscle groups over the hip, knee, and foot joints. We measured maximum torque as best of three trials. We assessed pressure sensibility around the ankles by using monofilaments of different stiffness (increments: 0,4, 2, 4, 10, 300 g) on the lateral malleoli. Reaction time (RT) was tested on the laboratory computer using a custom-made program; at random time intervals between 5–10 seconds, a visual and audio cue was produced at which the participant had to push a button as fast as possible. The average of five attempts was used. The data of the sensory and strength screening and testing has been published in detail [19].

Force plate (Kistler, Switzerland) measurements were recorded for each participant while they performed 30 seconds of quiet stance. First with eyes open, then with eyes closed. The signals were sampled at 3000Hz and filtered using a Butterworth low pass filter with a 10 Hz cut-off.

### Analysis

**Fig 1:**
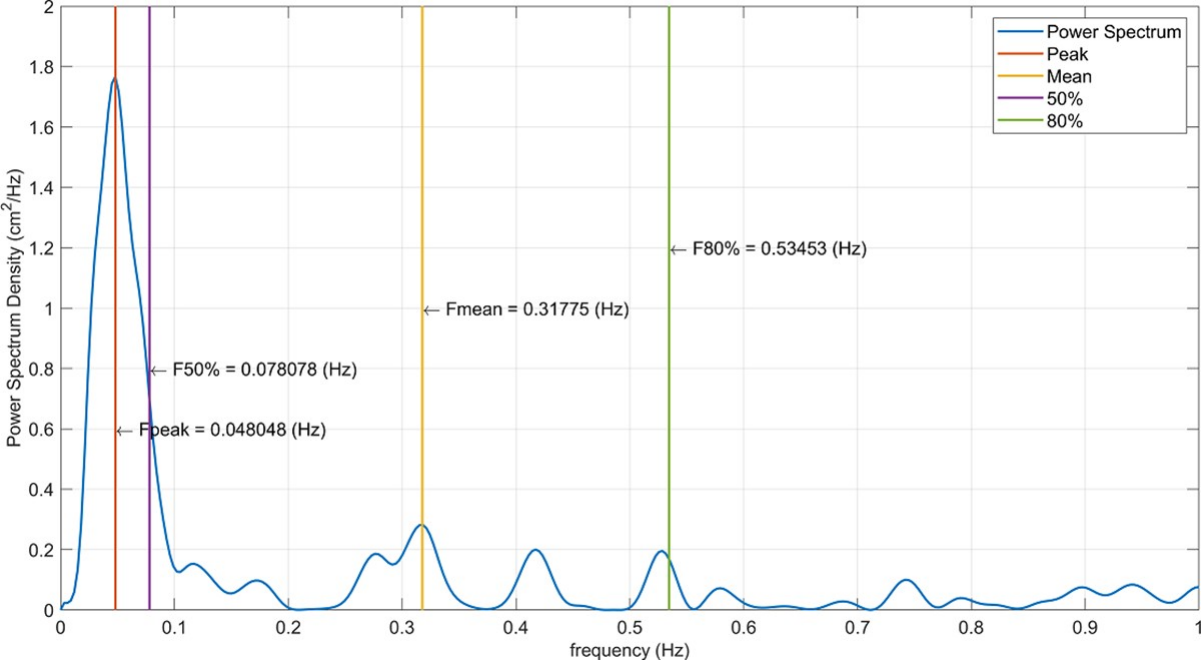
Example of a power spectrum density graph. The participant was standing quiet for 30 seconds on a hard surface with eyes open. The four extracted features are indicated by the vertical lines (see figure legend) and represent the frequencies at which peak and mean density were observed as well as the frequencies at which the area under the curve reached 50% and 80% of max power.

The CoP signal power spectrum density (psd) and its features (peak, mean, 50%, and 80%) were estimated using the Lomb-Scargle function in MatLab (Fig 1). The Lomb-Scargle, rather than a regular psd is more suitable to analyse low frequencies in relatively short time series [44]. To be able to cluster the participants based on the multifactorial systems underlying our questions, rather than a single variable, a PLS-tree (partial least squares) was calculated. This was done twice; once with a Y-block (dependent variables) of the psd features from open eyes quiet stance and once with a Y-block of the psd features from closed eyes quiet stance. Apart from nystagmus being excluded for the eyes closed analyses due to not having sufficient data points, the X-block (independent variables) was the same in both PLS-trees and consisted of the participants’ sensory and strength measures as well as their FES-I scores and reaction times. The groups found in the PLS-tree were then entered as classes in an OPLS-DA (orthogonal projection of latent structures–discriminant analysis) with the same X and Y blocks as their respective PLS-trees. (O)PLS is a multi-variate regression method that allows for several aspects that can be problematic in other methods: small number of observations versus the number of independent variables, noisy data, high covariance and most importantly more than one dependant variable [45]. As the motor control system is a complex system with many covariates, an (O)PLS based method to be able to discriminate between those with and without problems in that system combined with FrCwas the most adequate. Knowing which variables contribute the most to that discrimination is valuable when developing interventions. The models were evaluated based on separation, component and model significance, permutation testing, and misclassification tables [45]. The PLS-trees and OPLS-DA analyses were performed in SIMCA 14 (Umetrics, Umeå). Significant differences between trials (for the frequency features) and between groups (for group characteristics) were assessed with two-tailed t-tests.

## Results

The power spectrum density features of the total sample as well as the groups within each trial are presented in Table 2. Of the 3 individuals with a positive finding for nystagmus, 2 were not able to perform the SEC task. This made the sample size for the SEC trial 43 instead of 45 and also reduced the variance of the nystagmus variable in such a way that it had to be excluded in the modelling for that trial.

**Table 2:** Power spectrum density features.

Trial / group	Peak Hz	Mean Hz	50% Hz	80% Hz	FES-I	SPPB
SEO (n = 45)	0,069	0,300	0,160	0,480		
Group SEO1 (n = 28, 10 women)	0,078	0,337	0,180	0,538	20	10,9
Group SEO2 (n = 17, 17 women)	0,056	0,248	0,133	0,401	23*	9,8
SEC (n = 43)	0,117	0,403	0,271	0,685		
Group SEC1 (n = 23, 8 women)	0,165	0,450^†^	0,345^†^	0,778^‡^	18	11,3
Group SEC2 (n = 20, 19 women)	0,060	0,348^†^	0,186	0,582	24**	9,8*

SEO: Quiet stance eyes open, SEC: Quiet stance eyes closed. FES-I: Falls-efficacy scale international. SPPB: Short physical performance battery. The frequency features have been tested for means differences between trials only (indicating strategy changes). The clinical tests (FES-I and SPPB) have been tested for means differences between groups only (indicating distinct groups for both psychological and physical factors).

† Significantly different from the same group in the eyes open trial (p < 0,05).

‡ Significantly different from the same group in the eyes open trial (p < 0,01).

* Significantly different from group 1 in the same trial (p < 0,05).

** Significantly different from group 1 in the same trial (p < 0,001).

### Postural control classification models and group differences

**Fig 2:**
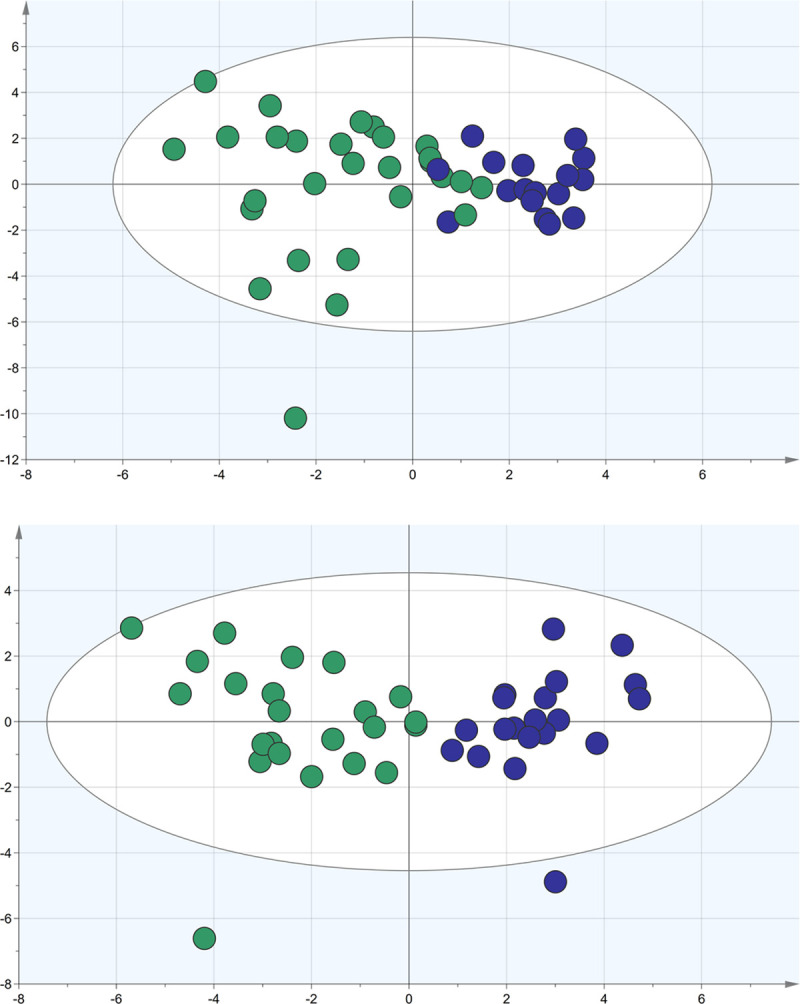
Group separation. Groups 1 are marked in green and groups 2 in blue. Top: Eyes open: shows some overlap. Bottom: Eyes closed: shows a clear separation.

Within each trial, the PLS-trees identified two groups, shown in Table 2 as SEO1/SEO2 and SEC1/SEC2. The eyes open classification model indicates that FES-I and the sensorimotor variables can explain the frequency-based grouping to 63% and predict it to 44%. The model is significant (p<0.001) and has good robustness but has poor separation (Fig 2). The eyes closed model (p<0.001), however, shows complete separation, can explain the frequency-based grouping to 66% and predict them to 63%. It is exceptionally robust and can classify members of the two groups with 100% accuracy (Fig 2). Even though the SEO model was strong with a moderate predictive value, there was some overlap between the groups, indicating prediction issues. Once the task become more difficult (SEC), those problems were gone. Some outliers emerged in the clustering (1 during eyes open and 2 during eyes closed). Upon further inspection these were natural outliers with slightly unexpected, but correct and verifiable values in some of the variables. Excluding them improved the models (not shown), but also falsely negated the large variance found in the actual population.

**Fig 3:**
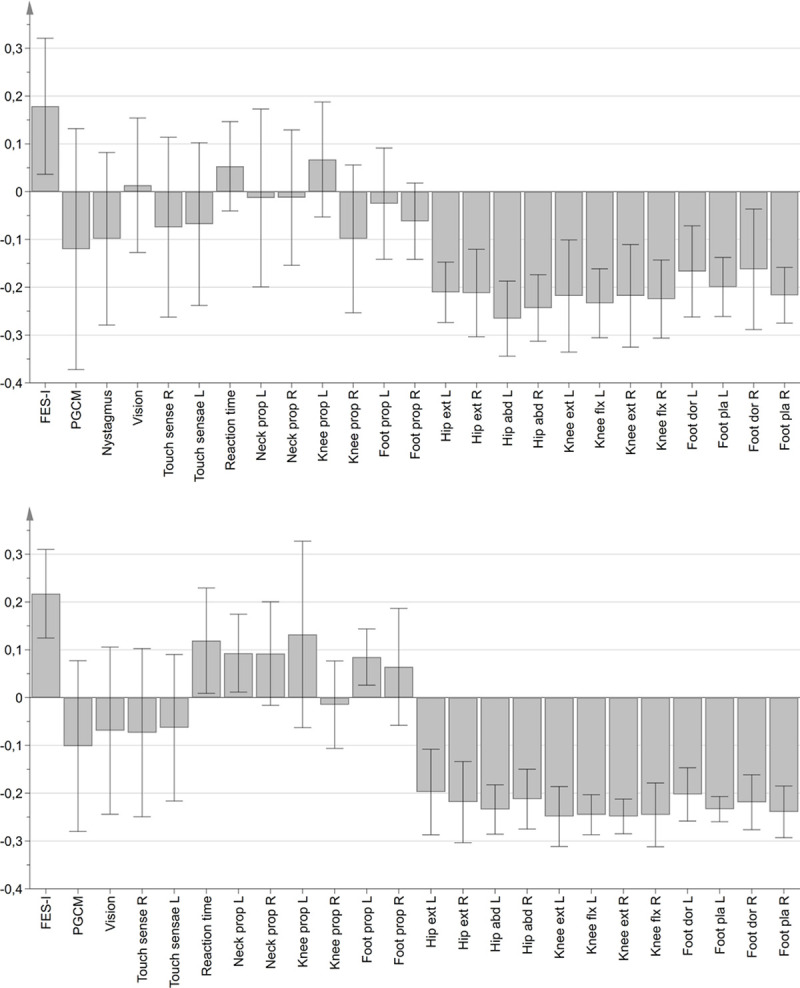
Variable contribution to the group discrimination. Top: Contribution of group 2 during eyes open quiet stance. Bottom: Contribution of group 2 during eyes closed quiet stance. Columns represent the (scaled) contribution of the variables to discriminate between groups 1 and 2 in each trial. The further the variable deviates from the total sample average (0 on the y-axis), the larger its contribution to the model becomes. Error bars not including 0 indicate significance for the particular variable.

When comparing the variable contributions to both models, we can see that with eyes open the largest contributions are by FES-I (p = 0,009), and muscle strength (p-values ranging from p = 0,03 for left foot dorsal flexion to p<0,001 for left hip abductors). When the groups are reformed based on the eyes closed trial data, FES-I variance becomes a larger contributor for group SEC2 than it was in SEO2. Alongside the FES-I and muscle strength variables, the contributions by reaction time (p = 0,02), neck (p = 0,05) and foot proprioception (p = 0,04) emerge as well when the participants have their eyes closed (Fig 3).

**Fig 4:**
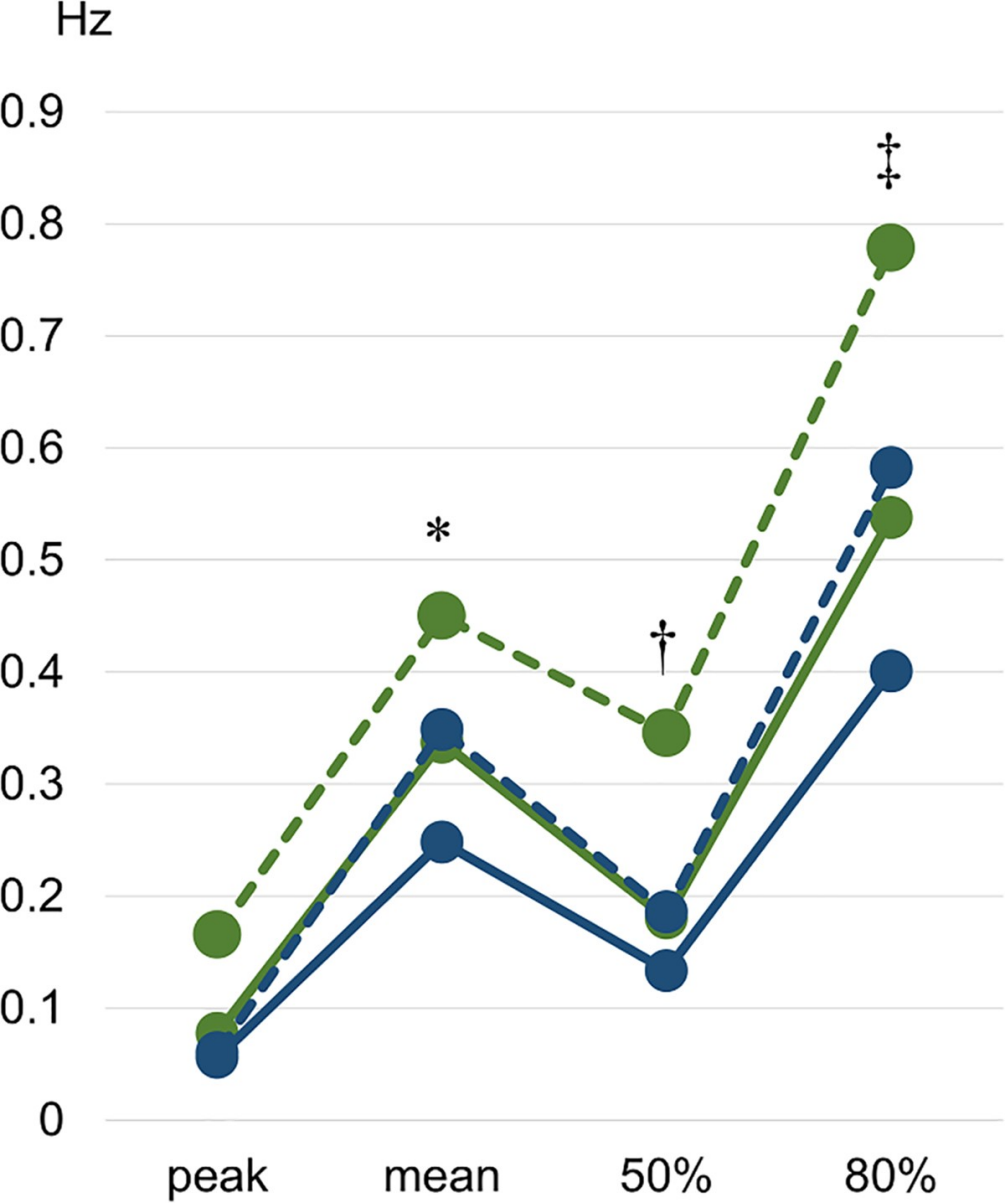
Power spectrum density features. Group averages for peak power, mean power, and 50% and 80% of the area under the psd curve. Solid lines: quiet stance eyes open trial (SEO). Dashed lines: quiet stance eyes closed trial (SEC). Green: group 1. Blue: group 2. * Both groups significantly different from the same groups in the eyes open trial (p < 0,05). † Only group 1 significantly different from the same group in the eyes open trial (p < 0,05). ‡ Only group 1 significantly different from the same group in the eyes open trial (p < 0,01).

When the task was more difficult due to imposed sensory disturbance (SEC, dashed in Fig 4), the density features of the power spectrum moved to higher frequencies; more so for group SEC1 than group SEC2. Both groups 2 (blue in Fig 4), which are the groups experiencing more concerns and more physiological decline, displayed their psd features at lower frequencies than group 1 within the same trial.

Group compositions of SEO1 and SEO2 were very similar to the compositions of SEC1 and SEC2, but not identical. Six participants belonged to group 1 in the eyes open trial, but group 2 during eyes closed. It was the other way around for 3 other participants (belonged to SEO2 but SEC1). As the individuals were harder to classify during the SEO trial, we can assume the groupings during the SEC trial to be more telling about group member features and control implications. The 3 who changed from SEO2 to SEC1 had very low FES-I scores (16–18) but were also bilaterally weaker in hip abduction and extension than the total sample average. Whereas the 6 who changed from SEO1 to SEC2 were only slightly more concerned about falling, were generally weaker, but also showed larger errors in all (but right knee) proprioception as well as a slower reaction time. The change from group 1 to group 2 upon closing their eyes indicates that eyesight is a strong compensator for declining sensory and motor systems. We will discuss this further in the next section.

## Discussion

We set out to explore the frequency domain of ageing postural control in quiet stance sway and discovered that those who show more decline in sensory and motor systems and a higher degree of FrC show more power in the lower frequencies of quiet stance. At first, this was unexpected as there is some indication that stiffness control strategies, which are expected to be utilised more during uncertain sensory input [46], are hypothesised to produce more power in higher frequencies. However, describing the slower and faster components of quiet stance CoP excursions as exploratory and reactive control respectively [23,24], could mean that those who are more concerned and more declined in their sensorimotor systems increase the potential of the exploratory aspects of control in order to gain more informative input to the system. This is also in line with the idea that movement variability is a tool to increase information to the system rather than a result of a failing system [47]. Another aspect is that visual feedback has previously been linked to lower frequencies in CoP due to a slower feedback loop [28]. In our sample this means that those who are more afraid and more declined in sensory and motor systems, depend more on vision and search for more sensory input than those who are not.

In addition, we found that when a change in control strategy might be warranted (closed eyes), those who show more decline in sensory and motor systems and more FrC do not shift the power density features to higher frequencies as much as those who do not experience any decline or concern. With the knowledge that the higher frequencies of CoP show very small and fast CoP deviations and reactions [48], we can assume that a decline in sensory systems might prevent the individual from registering small deviations. The usual reflex based reactive control is not getting triggered the way it should, which results in a non-reaction, in turn allowing for a larger, low frequency deviation before a reaction is produced. Therefore not only does a control strategy that is highly dependent on visual input show up as a low frequency sway output, a successful reweighting towards the other control systems after a loss in visual input shows up as a higher frequency sway output. In other words: merely a loss of visual input, raises the frequencies only a small amount, but successful reweighting raises the frequencies significantly. No participant in group SEC2 fell during the trials, indicating their strategy is not unsuccessful. Their strategy is one that has been adapted to the loss of adequate input to the system, which has led to reweighting issues.

As mentioned before, the SEC2 group was significantly more concerned about falling. Even though this study is of a cross sectional nature, we did get an interesting indication from the 6 individuals who were re-classified between trials from SEO1 to SEC2 that should be investigated further. This small sub-group showed declines in muscle strength, proprioception, and reaction time, and while those are highly correlated to increased FrC [19], they were not significantly more concerned about falling than group SEC1. This could suggest that as long as the visual compensation of sensorimotor decline works adequately, the individual does not develop FrC. Longitudinal studies concerning possible causality in this matter are required to further investigate this indication.

There seems to be a distinct difference between men and women. This difference has been clear in studies on FrC [16,49] as well as studies on balance and postural control [50,51]. In the current study we see that groups SEO2 and SEC2 consist mainly of women. With older women being generally more concerned and generally more declined in muscle strength than older men, that is not unexpected. Recreating the models with only women included, however, creates the same robust models with similar patterns of variable contribution and correlations (not shown). Unfortunately, the groups were too small to maintain power and therefore the models for the entire sample were reported. In future research, when moving towards designing clinically usable classification models, it is therefore especially important to recruit a large enough number of participants to be able to analyse men and women separately.

In many fields that use psd as an analytical tool, peak power is considered to be the most important feature of the spectrum. Because we did not only want to differentiate between sensory modalities but were much more interested in differentiating between control strategies in relation to concern and physiological decline, peak power turned out to be the least telling of the four features that were used. Peak power did not show significant differences between any of the trials or groups, therefore the other power features were needed as well. As postural control is about the ability to compensate and adjust strategy in order to adept to intrinsic and extrinsic factors, changes in the pattern of the entire spectrum should be the focus of future research in this area.

## Conclusions

During quiet stance, an increase in fall-related concerns and a decline in lower limb strength are strongly correlated with utilising a more explorative (slow) postural control strategy. The same aspects, together with reduced neck and foot proprioception and delayed reaction time, are also correlated with a difficulty to adequately activate more reactive (faster) postural control strategies when needed (eyes closed). Indications were found for compensatory vision being a protective strategy associated with the development of fall-related concerns, but larger longitudinal studies are needed to investigate this.
